# Corpus-based typology: applications, challenges and some solutions

**DOI:** 10.1515/lingty-2020-0118

**Published:** 2021-03-30

**Authors:** Natalia Levshina

**Affiliations:** Max Planck Institute for Psycholinguistics, Wundtlaan 1, 6525 XD Nijmegen The Netherlands

**Keywords:** analyticity, comparable corpora, corpus annotation, language comparison, parallel corpora, universals

## Abstract

Over the last few years, the number of corpora that can be used for language comparison has dramatically increased. The corpora are so diverse in their structure, size and annotation style, that a novice might not know where to start. The present paper charts this new and changing territory, providing a few landmarks, warning signs and safe paths. Although no corpus at present can replace the traditional type of typological data based on language description in reference grammars, corpora can help with diverse tasks, being particularly well suited for investigating probabilistic and gradient properties of languages and for discovering and interpreting cross-linguistic generalizations based on processing and communicative mechanisms. At the same time, the use of corpora for typological purposes has not only advantages and opportunities, but also numerous challenges. This paper also contains an empirical case study addressing two pertinent problems: the role of text types in language comparison and the problem of the word as a comparative concept.

## 1 Aims of this paper

Over the last few years, the number of corpora that can be used for language comparison has dramatically increased. The corpora are so diverse in their structure, size and annotation style, that a novice might not know where to start. The present paper is an attempt to chart this new and changing territory, providing a few landmarks, warning signs and safe paths.

Although some overviews and reflections on the use of corpus data in typology have been published already, they focus on specific corpus types. In particular, a special issue of Linguistic Typology and Universals (STUF) in 2007, edited by Michael Cysouw and Bernhard Wälchli, was the first systematic reflection on the use of parallel corpora in typology (Cysouw and Wälchli 2007). Comparable web-based corpora from the Leipzig Corpora Collection were discussed by Goldhahn et al. (2014). With new multilingual resources and corpus-based studies mushrooming in recent years, it is high time to provide an up-to-date overview of diverse corpus types (parallel, comparable and others) and show how they can be used for the purposes of large-scale language comparison and testing of typological generalizations. Note that contrastive linguistics, which compares a small number of languages in a highly detailed way, is beyond the scope of this paper. I also do not discuss here the role of typological information in development of NLP applications (see Ponti et al. 2019).

An overview of relevant corpora, including ongoing projects, is presented in Section 2. It seems that no corpora at present can replace the traditional type of typological data based on language description in reference grammars – at least, not for all research questions. Some fundamental linguistic properties, e.g. the presence of a definite article or the number of cases in a language, are much easier to find in a grammar than to extract from a corpus. The practicality of using grammars, however, comes at the cost of having to rely on someone else’s judgements and intuitions based on their own more or less representative corpora. Using corpora from the very beginning will allow us to investigate language use more directly and in greater detail (although corpora, of course, also represent the result of someone’s choices, from sampling to word segmentation and annotation).

In addition, corpora have been used successfully in many situations, which are listed below.

– In some cases, one can use corpora to enhance and complement traditional typological resources. See Section 3.
– Corpora can give us fine-grained information about the use of constructions in different contexts. This aspect is discussed in Section 4.
– Corpora can be used for obtaining quantitative measures that capture gradient properties of a language (e.g. analyticity or morphological complexity). See Section 5 for more detail.
– Corpora can be used for establishing new cross-linguistic generalizations in the form of intra-linguistic correlations or biases towards a particular strategy. They can also help to rethink already established universals (see Section 6).
– Corpora are indispensable if we want to detect common patterns in human interaction and speech production, including information density, speech rate, introduction of new referents and the use of scaffolding constructions (see Section 7).
– Corpus evidence can explain universals based on general cognitive and social pressures (see Section 8).
– Finally, corpora have been used to compare languages across time and space in diachronic and areal typology, and to reveal how typological characteristics of languages influence child language acquisition (see Section 9).

The use of corpora for typological purposes presents not only advantages and opportunities, but also numerous challenges. These are discussed in Section 10. Finally, Section 11 contains an empirical study demonstrating the use of corpora for language comparison. It shows to what extent the computation of analyticity indices depends on the choice of text types and on our decision about what to count as a word.

## 2 An overview of existing corpora

### 2.1 Parallel corpora

Multilingual corpora differ in the degree of similarity between the texts in every individual language. Parallel corpora, which are composed of aligned sentences or other chunks of text in two or more languages, display the highest semantic and pragmatic similarity between the components. The most “massively parallel” corpus up to date is the collection of New Testament translations with aligned verses (Mayer and Cysouw 2014). It contains more than 1850 translations in languages represented by more than 1,400 different ISO 639-3 codes.1Available on request from the authors. The corpus of the Universal Declaration of Human Rights contains 372 versions of this document in different languages or language varieties (Vatanen et al. 2010).2http://research.ics.aalto.fi/cog/data/udhr/.

Another large collection is the Opus corpus (Tiedemann 2012), which has very diverse texts – from documentation to news and from film subtitles to the European Parliament transcripts.3http://opus.nlpl.eu/. The number of languages for which aligned texts are available simultaneously is very different for different texts, which makes it difficult to use this collection for large-scale language comparison. There is also a bias towards European languages for many text types.

As for medium-size corpora, there is the Parallel corpus for Typology (ParTy), which contains film subtitles in most European languages and in some major languages of the world, such as Arabic, Chinese, Hebrew, Hindi, Japanese, Korean, Thai, Turkish and Vietnamese (Levshina 2015).4https://github.com/levshina/ParTy-1.0. Only caption text is available. Alignment between the sentences in different languages is automatic (read: not perfect) and based on the timing information in subtitles. The corpus ParaSol (von Waldenfels 2012) contains translations of fiction and some other genres in Slavic and other (Indo)-European languages.5http://www.parasolcorpus.org/. Most texts are lemmatized and have morphosyntactic tags.

Another parallel corpus with texts of the same type is Europarl, which has been used extensively for the purposes of Natural Language Processing (Koehn 2005).6http://www.statmt.org/europarl/. It was extracted from the proceedings of the European Parliament, including information about the speaker. It currently contains 20 languages, each aligned with English. The above-mentioned Opus corpus provides an interface for searching for translation correspondences in multiple languages.

Of particular interest for areal typologists is the collection of parallel texts in the languages of the Circum-Baltic area (Estonian, Finnish, Latvian, Lithuanian, Polish and Swedish), which are aligned with Russian as a part of the Russian National Corpus (Sitchinava and Perkova 2019).

Many researchers have used translations of popular books for language comparison. On the larger side one should mention A. de Saint-Exupéry’s *Le Petit Prince* translated into about 300 languages worldwide, used by Stolz et al. (2017). Some corpora include only genetically related languages from the same family or genus, e.g. the collection of translations of *Alice*’*s Adventures in Wonderland* and Paolo Coelho’s *Alchemist* in Indo-European languages by Verkerk (2014).7Another private parallel corpus is the Amsterdam Slavic Parallel Aligned Corpus created by Adrian Barentsen (p.c.). Because of copyright issues, corpora of contemporary fiction are usually not open access, but can be shared upon request.

### 2.2 Comparable corpora

Comparable corpora are those in which texts in different languages are not parallel, but represent similar text types or topics. For example, the Multilingual Corpus of Annotated Spoken Texts (Multi-CAST) contains spoken traditional and autobiographic narratives in eleven Indo-European and Oceanic languages (Haig and Schnell 2016).8https://multicast.aspra.uni-bamberg.de/. The data are limited, but richly annotated, including referent tracking. At the other end of the continuum (no annotation, but many languages) is the Leipzig corpora collection (Goldhahn et al. 2012), which contains web data, online news and/or Wikipedia articles in about 250 languages. The data are freely downloadable as lists of randomized sentences.9https://wortschatz.uni-leipzig.de/en/download.

For cross-linguistic child language acquisition research, one can use the CHILDES collection.10https://childes.talkbank.org/. One of the main bottlenecks in this research area is the data scarcity problem, exacerbated by the fact that the data are often not publicly available. A solution here is to select the languages with maximum diversity regarding the most relevant typological features (Stoll and Bickel 2013). This approach has been implemented in the ACQDIV database (Jancso et al. 2020), which represents fourteen typologically diverse languages. Most of the corpora are morpheme-segmented and annotated for parts of speech.

One should also mention here some ongoing projects. The Language Documentation Reference Corpora (DoReCo) represent personal and traditional narratives in more than 50 languages (mostly lesser-documented ones), each with a minimum of 10,000 words (Paschen 2020).11http://doreco.info/. They are time-aligned at the phoneme level. Some corpora are morphologically annotated, with POS tags, morpheme breaks and glosses. Another important example is the MorphDiv project at the University of Zurich, which aims to collect corpora of diverse genres representing 100 languages, so that it can be used for large-scale typological investigations.12Project “Non-randomness in Morphological Diversity: A Computational Approach Based on Multilingual Corpora”, https://www.spur.uzh.ch/en/departments/research/textgroup/MorphDiv.html. These corpora are currently under development and will become available in the near future.

### 2.3 Unified annotation

Some collections focus more on unified annotation and processing tools than on the similarity of texts representing different languages. A prominent example is the Universal Dependencies (UD) corpora (Zeman et al. 2020).13https://universaldependencies.org/. At present (August 2020), they have more than 150 syntactically and morphologically annotated corpora (treebanks) in more than 90 languages. The texts are very diverse – from spoken data to Bible translations and from legal texts to social media. Their main advantages are consistent rich annotation and detailed documentation. One can also use the UD-related tools for annotation of new texts (see Section 11).

Unified multilevel annotation of spoken corpora, with a focus on semantic and pragmatic features of referential expressions, is available in the GRAID annotation schema (Haig and Schnell 2014).

Usually, there are trade-offs between the size of individual corpora (especially less well described languages), representativity of registers and text types, typological diversity and the information represented in annotation. None of the existing corpora have very high scores on all these dimensions. The typological diversity is the greatest in the Bible translation corpus, but it only represents one (quite specific) text type and contains no grammatical information. The UD corpora, in contrast, provide rich morphosyntactic and lexical information, but many fewer languages. In addition, some UD corpora, especially non-Indo-European ones (e.g. Tagalog, Telugu, Warlpiri and Yoruba), consist of very small text samples.

All this leads us to conclude that corpora are not yet ready to become the main source of data for mainstream typological research.14At least, directly, since many grammars are based on corpora collected during fieldwork. Unfortunately, these corpora are not always publicly available. However, existing corpora are useful and sometimes indispensable for numerous other purposes, which are discussed in the following sections.

## 3 Corpora as a source for traditional typological data

In some cases corpora can be used to extract typological data, or at least to enhance and complement them. For example, Stolz et al. (2017) extract interrogative spatial pronouns related to the location (*Where?*), goal (*Whither?*) and source (*Whence?*) from numerous translations of *Le Petit Prince.* Using the corpus data allows them to increase the number of languages in comparison with previous studies, obtaining 183 translations in different languages and language varieties. Together with other sources, this study covers about 450 languages and varieties in total. These data allow the authors to investigate how interrogative paradigms are distributed geographically and to detect areal patterns.

In lexical typology, parallel corpora can help to search for colexification patterns, i.e. distinct meanings for the same lexical item (François 2008), which are usually inferred from elicited wordlists and dictionaries (cf. online database of Cross-Linguistic Colexifications [CLICS] by List et al. 2018). For example, Ӧstling (2016) extracts three colexification patterns – STONE / MOUNTAIN, ARM / HAND, and TREE / FIRE – from 1,142 New Testament translations representing approximately 1,000 languages. This approach allows him to identify areal patterns. For example, the TREE / FIRE colexification is widespread in Australian languages, as well as in Papua New Guinea, which supports the findings in Schapper et al. (2016) based on more traditional typological data sources (grammars, dictionaries, wordlists and expertise of language specialists).

This method of data collection from parallel corpora will work best when there is a salient default variant and little intra-linguistic variation. For instance, English speakers ask about the location of an object by using *where* in most contexts and situations. In other words, this method works well when reference grammars and dictionaries work well, too. The grammatical function or lexical meaning should also be frequent enough in order to be found in a relatively small text. However, when using parallel corpora for this purpose, one should be prepared to deal with a lot of noise, due to alignment issues and idiosyncratic choices made by translators.

## 4 Corpora as a source of fine-grained and gradient information

Reference grammars are usually good for understanding the main strategy of expressing a certain meaning, but they can be less useful for describing less frequent, context-dependent ones. Moreover, this descriptive information does not reflect the gradient nature of language use (Levshina 2019; Ponti et al. 2019). This leads to data reduction and bias towards investigating categorical features with a bimodal cross-linguistic distribution (Wälchli 2009).

For example, word order is often described with the help of categorical labels. Languages can have one of the six possible combinations of SOV, SVO, VSO, etc. or no dominant order (Dryer 2013). With the emergence of multilingual corpora, one can measure the word order preferences directly (e.g. Östling 2015) and provide more precise numeric estimates instead of categorical labels, avoiding loss of information.

Similarly, Wälchli (2009) investigates the word order of verb and locative phrases in imperative constructions in 100 translations of the Gospel of Mark into languages from all continents. He shows that the proportions of V + Loc versus Loc + V are higher in imperative contexts in comparison with the non-imperative contexts. It would be difficult or impossible to obtain this information from grammars.

Another example is causative constructions. Grammars have a bias towards describing only the “default” causative, which expresses the prototypical causation: direct, factitive (i.e. non-permissive) and implicative causation (e.g. *break X*, *make X cry*). At the same time, peripheral functions, such as forceful, accidental, permissive, assistive, curative and non-implicative causation (e.g. order X to do Y), are often ignored. Also, the semantic labels are often inconsistent and vague, so that it is difficult to compare the constructions cross-linguistically. Parallel corpora can provide an important source of evidence in this case, giving a fine-grained picture of form-meaning mappings in different languages (Levshina, In press).

When a typologist is interested in gradient and context-dependent features, he or she will end up doing variational linguistics. In order to model the use of linguistic variants properly, as in the Labovian tradition, one needs a substantial amount of text data, which can be analyzed with the help of multivariate methods, such as regression analysis or classification and regression trees (CART). This approach is extremely labour intensive, and hardly feasible for a representative number of languages. To save time and effort, one can use an advantage provided by a parallel corpus. More exactly, one can code the semantic and pragmatic features (the independent variables) only in the source text, and analyse the linguistic variants (the response variable) in the translations (see examples in Levshina 2016, 2017a).

## 5 Corpora as data for obtaining new descriptive typological measures

### 5.1 Analyticity, syntheticity and other corpus-based indices

The classification of languages into isolating, agglutinative, fusional (inflectional) and polysynthetic goes back to the works by August Wilhelm von Schlegel (1818), Franz Bopp (1816) and Wilhelm von Humboldt (1822). Despite criticisms (e.g. Haspelmath 2009), it has been very influential in the history of linguistics and remains widely used today (cf. Croft 2003: 45).

The indices describing these parameters can be derived from corpora. Greenberg (1960) formalized them for the first time for quantitative morphological typology. He created a list of indices, such as the index of synthesis (the ratio of the number of morphemes to the number of words in a text sample), the index of agglutination (the ratio of agglutinative constructions to the number of morpheme junctures), suffixal index (the ratio of suffixes to the number of words) and others. Most of these indices are a corpus-based implementation of Sapir’s (1921) typological criteria. The complete list is provided in the Appendix. The indices were computed manually by Greenberg on the basis of 100-word samples of text.

With the advances of new annotated corpora, the procedure of data extraction becomes easier. In particular, Szmrecsanyi (2009) computed automatically the indices of analyticity and syntheticity for samples from diverse English corpora. One can easily estimate analyticity on the basis of UD annotation of functional and content words (see Section 11). Evaluation of syntheticity and other morphological parameters is, however, more problematic because we do not have corpora with morpheme segmentation (although see the DoReCo project in Section 2.2) or reliable tools for automatic morphological analysis, especially for non-Indo-European languages. One should also mention here the dependence of these indices on text types and the definition of the word as the main unit of analysis. These issues are discussed in Section 11.

### 5.2 Information-theoretic approaches to grammatical complexity and lexical diversity

The question of linguistic complexity has attracted a lot of attention (e.g. Dahl 2004; Hawkins 2004; Hockett 1958, to name just a few). There are many different approaches to measuring grammatical complexity: global (e.g. complexity of grammar in general) and local (e.g. complexity of the tense and aspect system); one can also focus on different criteria, such as the presence of overt coding, variability of grammatical marking or system dependencies (for an overview see Sinnemäki 2014).

Complexity measures have been derived from corpora using concepts from information theory. These measures usually describe morphological or word order complexity as a global property. In particular, Juola (1998, 2008 used Kolmogorov complexity to measure the informativeness of a given string as the length of an algorithm required to describe/generate that string. Consider a simple illustration. A sequence *ababababab* is easy to describe, whereas a sequence *abaabbbaba* is more difficult because there is no systematic pattern that would allow us to predict which symbol comes next.

Kolmogorov complexity cannot be computed directly because it is computationally intractable, but it can be approximated by using a simple ZIP file compressing program. Suppose we have a file with 10,000 characters, which represent a regular sequence *abababab*…. The size of this file is 10,000 bytes. We also have a file of the same length, which contains a sequence of *a* and *b* in random order, e.g. *aababbbaa*… Its size is 10,000 bytes, as well. When the file with the first, regular sequence *abababab*… is compressed using a compression algorithm, the size of the archive is only 211 bytes. We see a huge reduction. When we use the same algorithm to compress the file with the random sequence, the size of the archive is 1,744 bytes. The compression rate of the random sequence is much lower, which means that its complexity is higher.

When estimating a specific type of grammatical complexity, an extra step is usually made, which involves some distortion of the original text. For example, if one is interested in the complexity of the inner structure of words (as a proxy for morphological complexity), one can randomly remove a fraction of characters or reshuffle them. Alternatively, one can replace all wordform types with a unique number (e.g. *walk – walked – walking* as 10, 98, 5 and *love – loved – loving* as 84, 25 and 67), which makes the language appear maximally suppletive, as if all wordforms are irregular. If one wants to focus on word order complexity, one can remove a fraction of the words or reshuffle them. The last step is to zip the file. Then one can estimate how much weight this type of complexity has in the total complexity by computing the ratio of the original compressed file size to the distorted compressed file size. Languages with complex morphology or with rigid word order will have a higher ratio because the predictable morphological and syntactic patterns will be lost in the distorted files.

According to Juola (1998), morphologically rich languages like Russian and Finnish have a relatively high ratio (above 1), whereas morphologically poor ones like Maori and English have a lower ratio (less than 1). The procedure is based on the substitution of each wordform in the text with a random symbol, i.e. the suppletivization method described above.

In addition to simple compression ratios, one can also compute compression distances between corpora in different languages. Two corpora are considered close if we can significantly “compress” one given the information in the other. Using compression distances based on the translations of the Universal Declaration of Human Rights in diverse languages, Li et al. (2004) and Cilibrasi and Vitányi (2005) create phylogenetic trees, which reproduce the genealogical classification (see also Benedetto et al. 2002).

An important question is which corpora are the most suitable for this kind of comparison. Sometimes it is recommended to use a corpus with the same message, i.e. a parallel corpus. Interestingly, Juola (1998) demonstrates that the size of the compressed New Testament translations in 6 different languages (Dutch, English, Finnish, French, Maori, and Russian) is approximately the same, although the original files are very diverse in their size. However, as was demonstrated by Ehret and Szmecsanyi (2016), this approach can be extended to semi-parallel and even non-parallel corpora. They computed morphological and syntactic complexity scores of different versions of the Bible and *Alice*’*s Adventures in Wonderland*. They also took newspaper texts in several European languages and in different varieties of English. Their distortion method involved removal of 10% of characters or words, depending on the type of complexity. In all types of corpora, the ranking of the languages was highly similar.

Importantly, one can test correlations between different complexity scores. For example, Ehret and Szmrecsanyi (2016) found a negative correlation, or a trade-off, between syntactic and morphological complexity. This trade-off was also demonstrated by Koplenig et al. (2017) on a much larger sample of 1,200 typologically and genealogically diverse languages from all parts of the world. Their methodology involved reshuffling characters or words. They also use entropy scores based on minimum substring lengths, instead of zipping the files. The different methods and data sources thus converge, revealing a similar pattern: the more information is carried by word order, the less information is conveyed by the internal structure of a word, and the other way round.

One should also mention here studies of lexical diversity, i.e. the abundance of word types in relation to their token frequencies. Bentz (2018) computes unigram entropy as a proxy for lexical diversity using Bible translations in more than 1,200 languages. His analyses demonstrate that lexical diversity decreases with the increasing proportion of L2 learners of a given language.

### 5.3 Word order flexibility and entropy

Corpora are indispensable if one wants to study word order flexibility. This concept can be quantified with the help of Shannon’s entropy. In its simplest form, entropy *H* of a binary word order contrast is maximal (*H* = 1) if two possible orders, e.g. VO and OV, occur with equal frequency (50/50). Entropy is minimal (*H* = 0) if only one order is possible (either VO or OV). Levshina (2019) uses entropy for different syntactic dependencies (e.g. Verb – Object, Adjectival Modifier – Noun) and co-dependents belonging to the same head (Subject – Object and Object – Oblique) from the Universal Dependencies corpora and Leipzig Corpora Collection. The results reveal a range of *H* values, from nearly 0 (no variation) to almost 1 (the 50/50 case). Regression analysis is used to explain this diversity by such factors as formal confusability of Subject and Object, frequency and function of the dependents.

It is an open empirical question to what extent word order entropy depends on text types. The results seem stable across different web-based registers (Levshina 2019), but a more detailed check on more diverse text types and registers, especially spoken data, is needed.

Another important question is how much data one needs in order to obtain stable reliable measures. Futrell et al. (2015) use diverse non-lexical information, conditioning the entropy scores on such features as relation type, part of speech of the head and dependent, and syntactic subtree (a head and its immediate dependents). Their most detailed metric, called Relation Order Entropy, however, depends on corpus size because of data sparseness issues. They conclude that the method “does not seem to be workable given current corpora and methods”. Simpler coarse-grained measures are more stable. For example, one only needs about 500 sentences in order to obtain stable measures of word order entropy of basic constituents (Allassonnière-Tang 2020).

## 6 New and updated typological generalizations

### 6.1 Cross-linguistic and intra-linguistic correlations

Linguistic universals come in different flavours. If the relationship between two typological parameters is bidirectional, we speak of typological correlations. For example, languages with OV are predominantly postpositional, whereas languages with VO are predominantly prepositional (Dryer 1992; Greenberg 1963). Corpora can also be used for identification and testing of typological correlations. For instance, word order correlations between verb-headed and noun-headed dependencies in the UD corpora have been tested by Guzmán Naranjo and Becker (2018). Another example is the negative correlation between word-internal and word order complexity, which was mentioned in Section 5.2. In contrast to traditional typological investigations, corpus-based correlations involve continuous estimates of typological parameters, such as the proportion of a particular word order or an information-theoretic complexity measure.

In these cross-linguistic correlations, every language represents one data point. In addition, correlations can be computed based on individual units within a language. The best-known example is probably the correlation between frequency and length of linguistic units, known as Zipf’s Law of Abbreviation (1965[1935]): more frequent words tend to be shorter than less frequent ones. Using Bible translations and the Universal Declaration of Human Rights, Benz and Ferrer-i-Cancho (2016) found a negative correlation between word length and frequency in a huge sample of almost a thousand languages from 80 diverse language families. They conclude that Zipf’s Law of Abbreviation is an “absolute” synchronic universal, in the sense that it is found in every language. At the same time, it only represents a statistical tendency at the level of an individual language. It has also been argued that average predictability of a word from its previous context as *n*-grams is more strongly correlated with word length than simple frequency (Piantadosi et al. 2011). For computation of predictability measures, it is convenient to use *n*-gram datasets, such as the Google web dataset with *n*-grams in 10 languages (Brants and Franz 2009).

Similarly, Stave et al. (In press) demonstrate on the basis of morphemically annotated corpora of typologically diverse languages (see the DoReCo project in Section 2.2) that Zipf’s Law of Abbreviation also holds for morphemes. In addition, they find that the mean length of a morpheme in a multimorphemic word correlates negatively with the number of morphemes in the carrier word where this morpheme appears, as well as with the carrier word length. These findings support Menzerath’s law, which runs as follows: the longer the superordinate unit, the shorter the units that constitute it (Altmann 1980; Menzerath 1954).

### 6.2 Typological implications

Another type of cross-linguistic generalization is implicational universals, which posits a one-way relationship between features. Corpus-based studies rarely address implicational relationships, focusing instead on correlations and biases. However, corpus data can be used to obtain additional insights about implicational relationships. An example is Gerdes et al. (2019), who investigate Greenberg’s Universal 25, “If the pronominal object follows the verb, so does the nominal object” (Greenberg 1963). This is an implication because it works only in one direction: if the nominal object follows the verb, the pronominal object may or may not do the same.

**Figure 1:**
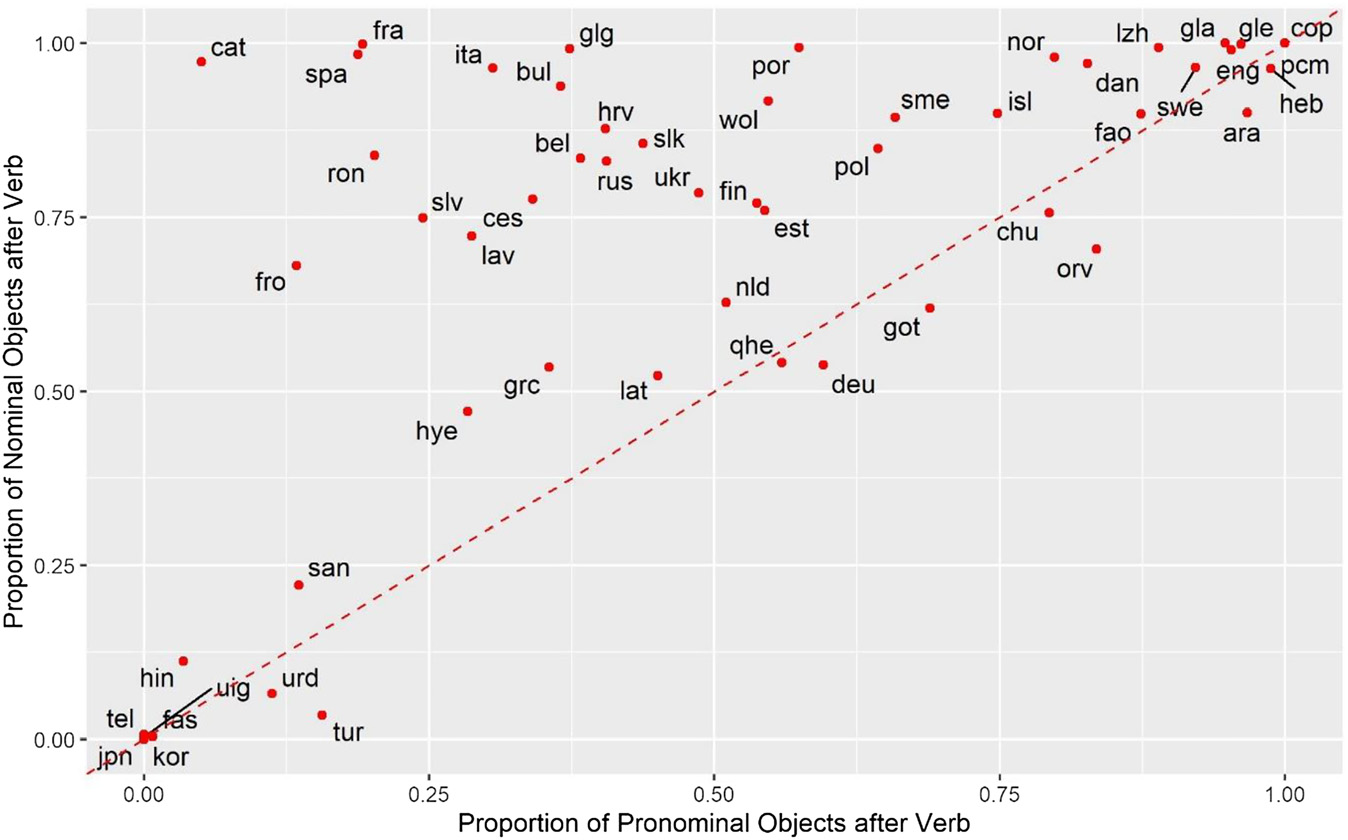
Proportions of pronominal objects (horizontal axis) and proportions of nominal objects after verbs (vertical axis) in the Universal Dependencies corpora (version 2.6). The labels represent ISO 639-3 codes.

Figure 1 displays the proportions of nominal and pronominal objects after the lexical verb in the Universal Dependencies corpora (version 2.7, Zeman et al. 2020).15Only corpora with at least 50 pronominal objects and 50 nominal objects (common nouns) were taken into account. The objects were counted in main clauses only. The counts are averaged across different corpora representing the languages. The data are available in Supplementary Materials. Every dot represents a language. The Hindi (hin), Japanese (jpn), Korean (kor), Persian (fas), Telugu (tel), Turkish (tur), Urdu (urd) and Uyghur (uig) corpora, in which both pronouns and nouns precede the verb, are in the bottom left corner. Arabic (ara), Coptic (cop), English (eng), Hebrew (heb), Irish (gle), Scottish Gaelic (gla) and a Nigerian creole Naija (pcm), which have pronominal and nominal objects following the verb, are in the top right corner. The Romance languages (Catalan cat, French fra, Spanish spa) are in the top left corner, because they have preverbal pronominal objects and postverbal nominal objects. In full accordance with Greenberg’s generalization, we do not have any languages in the bottom right corner, where preverbal nominal objects and postverbal pronominal objects would be.

In addition, we observe that the lower triangle is nearly empty, although there are some exceptions. For example, the Turkish corpora (tur) have a few pronominal objects after the verb. An inspection of the sentences reveals that these are mostly emphatic contexts with pronominal objects, as *beni* “me” in the following example:

(1)

**Table:** 

*Artık*	*hiçbir*	*şey*	*avutamaz*	***beni***
now	no	thing.NOM	comfort.NEG.AOR.3SG	me.ACC
“Nothing can comfort **me** now.”				

This right dislocation is typical of backgrounded information, which can be predictable from the context or already given (Erguvanli 1984: 56–57). The motivation for this phenomenon seems to be the tendency to put more urgent and important information first (Givón 1991).

And yet, most of these languages are close to the border. On the basis of the data, one can formulate a quantitative probabilistic version of Greenberg’s universal: In a doculect, the proportion of pronominal objects in the postverbal position is not substantially higher than the proportion of nominal objects. But what does “substantially higher” mean? The maximal difference between the proportion of postverbal pronouns and the proportion of postverbal nouns is found for Old Russian (orv): 0.83–0.70 = 0.13. For Turkish, the difference is only 0.15–0.03 = 0.12. As a hypothesis, it is unlikely that the difference between the proportions will be greater than 0.20. This tendency can be explained by general processing principles, such as analogy and the tendency to put accessible and short constituents first (e.g. Hawkins 1994). This hypothesis should be tested on languages from different families and parts of the world.

In order to investigate the traditional typological implications or correlations, one needs more diverse corpus data with annotation labels that are compatible with the typological hypotheses. Often, this is quite difficult. For example, very few corpora have systematic annotation for demonstrative pronouns, descriptive adjectives or even declarative sentences – comparative concepts that play a role in Greenberg’s (1963) famous word order universals. Morphological structure and semantic classes are usually not available, either, although some projects, such as Multi-CAST and DoReCo, partly solve these problems.

### 6.3 Intra-linguistic biases

A bias here is a cross-linguistic tendency to prefer one linguistic feature to another or to have higher or lower values of a particular value than a certain threshold. For example, in the overwhelming majority of the world’s languages, subject appears before object (Dryer 2013).

**Figure 2:**
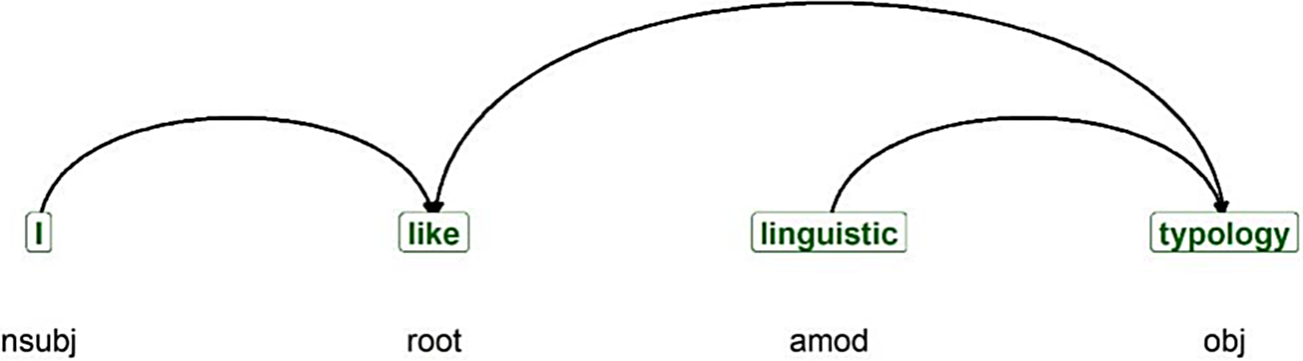
Example of a graph with syntactic dependencies.

With corpora, one can compute plenty of different values. For example, there is evidence that dependency distances between the head and the dependent tend to be smaller than one can assume by chance. The distances are computed as follows. Consider the illustration shown in Figure 2 with the sentence “I like linguistic typology.” The dependency distance between the root (the verb *like*) and the subject (*I*) is one word. The distance between *like* and *typology*, the object, is two words because the adjective *linguistic* intervenes between them.

The main idea is that dependency distances are on average shorter than one would expect based purely on chance (Ferrer-i-Cancho 2006; Futrell et al. 2015; Liu 2008). The reason is that long dependencies have higher memory and integration costs. One manifestation of this bias is the principle “short before long” (cf. Hawkins 2004), which minimizes the domains for recognition of constituents in VO languages.16It is not important whether we speak about dependencies or syntactic constituents in this context. The predictions are identical. Consider two versions of the same sentence below:

(2)

a.

**Table:** 

*I’ve read [your article on classifiers in Sinitic languages] [with great interest].*

b.

**Table:** 

*I’ve read [with great interest] [your article on classifiers in Sinitic languages].*

These versions differ in the order of constituents. In (2a), the object follows the verb, and the prepositional phrase PP comes later. In (2b), the PP comes first, and the object comes later. The version (2a) is less efficient than the version (2b) because the long object phrase *your article on classifiers in Sinitic languages,* which consists of seven words, substantially increases the distance between the verb and the PP *with great interest*, which consists of three words. In the Universal Dependencies annotation style, the sum dependency distances will be 31 words in (2a) and 27 in (2b). This means that the variant (2b) is more efficient and therefore should be preferred by language users.

This principle is not observed in all constructions and languages, however. Liu (2020), who compares the position of longer and shorter prepositional phrases relative to the head verb, shows that the tendency to put shorter PP closer to the verb is only strong when one compares pairs of postverbal PPs. If one takes preverbal PPs, which are possible in some SVO languages and are the default in head-final languages like Hindi/Urdu and Japanese, the tendency to put long before short, as one would expect from the principle of dependency length minimization, is not observed.

### 6.4 Common semantic dimensions

Another type of cross-linguistic generalization, which is less frequently discussed in the literature, is common semantic dimensions and contrasts that are expressed by distinct forms in most languages.

Parallel corpora are particularly well suited for induction of such dimensions. The contextual meanings that need to be expressed by translators into different languages provide convenient *tertia comparationis*. An example of this approach is Wälchli and Cysouw (2012), who use the *Gospel According to Mark* in 100 languages, and find that most languages make a distinction between the semantics of going and coming. Many translations also distinguish coming from arriving (e.g. the texts in Hungarian and Modern Spanish). Using similar methods, Levshina (2015) compares analytic causatives in a corpus of film subtitles in 18 European languages and concludes that the most typical distinction, which is expressed formally in the languages, is that between “making” and “letting”. This distinction emerges automatically as the first dimension on a Multidimensional Scaling map.

The corpus-based approach allows us to incorporate the information about the frequencies of particular functions, since some functions and meanings are common and some are rare. This is an advantage in comparison with the experimental approach to semantic maps (e.g. Majid et al. 2008). In situations where one has very many different semantic distinctions, and one cannot create an etic grid with all possible configurations of parameter values known, corpora can help to filter out untypical combinations, focusing only on frequent configurations relevant for language users’ experience.

The main problem with this approach is the fact that one has to rely on a specific doculect (e.g. the New Testament translations) with possible translationese effects (i.e. influence of the source text on the target text) and idiosyncrasies (e.g. archaic Bible translations may not reflect the choices made by the present-day language users). Therefore, any extrapolations from a doculect to other varieties and the “language as a whole” should be made with caution.

## 7 Speech and discourse universals

Corpora are indispensable if one wants to study cross-linguistic commonalities in speech and discourse. For example, extensive corpus data from different languages (e.g. Du Bois 1987; Du Bois et al. 2003) were used to formulate and test the principles of so-called Preferred Argument Structure:

– Avoid more than one lexical core argument (*One Lexical Argument Constraint*)
– Avoid lexical A (transitive subjects) (*Nonlexical A Constraint*)
– Avoid more than one new core argument (*One New Argument Constraint*)
– Avoid new A (*Given A Constraint*)

This line of cross-linguistic corpus research has contributed much to our understanding of how language structures accommodate to language users’ mechanisms of discourse processing and production.

Corpus data can also help to study constructions that scaffold human interaction. An example is Dingemanse et al. (2013), who argue that a word like “huh?” is a universal word. This word serves as a repair initiator, which signals that there is a problem in understanding, but leaves open what the problem is. Dingemanse et al. (2013) analyse natural interactions in 10 diverse languages, arguing that the cross-linguistic similarities in the form and function of this interjection are much greater than one would expect by chance.

Corpora are indispensable for studying speech and information rate. For example, a study by Coupé et al. (2019) based on a sample of spoken and written data from seventeen languages shows that average speech rate (the number of syllables per second) in a language is negatively correlated with information density, which represents how unexpected on average a syllable is, given the previous syllable in a word. The average information rate (information density multiplied by speech rate) is around 39 bits per second across the languages. This allows for efficient use of the communication channel. Another example is Seifart et al. (2018), who show that nouns slow down speech more than verbs do in all the languages they studied. That is, nouns universally require more planning, probably due to the fact that they usually introduce new information (otherwise, they are replaced by pronouns or omitted).

It would be impossible to obtain these and similar generalizations without corpus data. The downside of this approach is practical: spoken data collection, transcription and annotation are a daunting task. As a result, the diversity of corpora and their size remain quite limited.

## 8 Usage-based explanations of cross-linguistic generalizations

If we want to understand why some patterns are common across languages and some are rare, we need to know how they are used by speakers. An example is markedness phenomena. Greenberg (1966) used corpus counts in order to show that formally marked categories (e.g. plural nouns, future tense, adjectives in the positive form and ordinal numerals) are less frequent than unmarked ones (e.g. singular nouns, present tense, comparative adjectives and cardinal numerals).

More recently, Haspelmath et al. (2014) provided corpus data from five diverse languages to explain why some verbs usually take part in inchoative alternations (e.g. Russian *slomat’* “break.TR” > *slomat*’*sja* “break.INTR”), which create non-causal forms from causal ones, whereas other verbs usually take part in causative alternations (e.g. Finnish *sulaa* “melt.INTR” > *sulattaa* “melt.TR”), which produce causal forms from non-causal ones. Verbs participating in inchoative alternations have on average higher corpus frequency of the causal form, while verbs participating in causative alternations have higher frequency of the non-causal (inchoative) forms (cf. Samardžić and Merlo 2018). This represents an example of linguistic efficiency because more frequent meanings are expressed by shorter forms, and less frequent ones by longer forms (see also Levshina 2018).

Other examples include local markedness, e.g. singulative forms of Frisian and Welsh nouns that occur more often in pairs or groups than alone (Haspelmath and Karjus 2018; Tiersma 1982) and differential case marking (Levshina 2018). Frequencies are interpreted as the driving force for emergence of structural asymmetries.

A problem with this approach is that we usually have access to contemporary corpus data, not the data that might have been relevant for the emergence of the relevant structures many centuries or even thousands of years ago. The convergence of results based on different text types and languages, however, gives us reasons to hope that these results can be extrapolated across time and space. Ideally, since corpus data yield only correlations, these results should be supplemented by artificial language learning experiments, which would demonstrate that the universal communicative and cognitive pressures could indeed be responsible for common cross-linguistic patterns (cf. Fedzechkina et al. 2016; Kurumada and Grimm 2019).

## 9 Corpora and languages in diachronic and areal typology and language acquisition

Synchronic corpora can be used for diachronic and areal typology. In particular, we can infer which typological changes happened and at what speed. For example, Verkerk (2014) investigates the encoding of motion events in Indo-European languages, using translations of three novels. Since Talmy (1991), languages have been classified into verb-framed and satellite-framed, depending on how they encode motion events. The ancestral state estimation analysis with the help of phylogenetic methods indicates that Proto-Indo-European did not belong to any of the clear types, although it did have a slight tendency towards the satellite-framed end of the scale. The main challenge of this approach is the difficulty of falsifying the results, given that the data from the previous stages are usually not available. One also needs large phylogenetic trees in order to infer the past states. The Indo-European family is probably the only one for which sufficient diachronic and parallel corpus data are available at the moment.

Similarly, parallel corpora can be used for areal typology. For example, van der Auwera et al. (2005) use J.K. Rowling’s *Harry Potter and the Chamber of Secrets* translated into Slavic languages to identify areal patterns in the expression of epistemic possibility.

Finally, multilingual corpora of child language can be used to understand the universal patterns in child language acquisition and the role of typological properties of a language in this process. For example, Moran et al. (2018), who analyse corpora of child-surrounding speech in seven typologically diverse languages from the ACQDIV database (see Section 2.2), show that morphological information serves as reliable cues for learning syntactic categories of verbs and nouns in all these languages. The role of words as cues is language-dependent, however.

## 10 Taking stock: advantages, challenges and possible solutions

We have seen that corpora have been fruitfully used to answer old questions and formulate new ones. Corpus data are particularly well suited for investigating probabilistic and gradient properties of languages and particular constructions and for identification and interpretation of cross-linguistic generalizations from the processing and communicative perspectives. They can often provide a more objective picture than reference grammars containing compact, tidy and unavoidably simplified descriptions, which may lead the researcher to expect more homogeneity than in the reality. We can hope that corpora will bring typology forward by showing how reliable the mainstream approach is. At the same time, there are some challenges, as well as possible solutions, which are summarized below.

Challenge 1. *Eurocentrism and data sparseness*. Most collections have a strong bias towards (Indo-)European languages. The corpora of other languages are often very small or insufficiently annotated. It is nearly impossible to find a typologically representative sample of languages of decent size (e.g. several hundreds). At the same time, there are some ongoing projects at the moment whose aim is to provide large samples of annotated texts from typologically diverse languages (e.g. the DoReCo and MorphDiv projects mentioned in Section 2). Similarly, the UD corpora are constantly growing as new languages are added.

Challenge 2. *Restricted registers and text types*. In general, a corpus is never equivalent to “the” language. It can only represent a tiny fraction of the language users’ behaviour. Many registers and text types are underrepresented. We still have few spoken corpora, especially representing spontaneous communication. The corpora are often small with respect to the number of languages and the amount of text. Written language is better represented, but the number of genres is often restricted to web-based language (e.g. online news and Wikipedia articles) or Bible texts, which often represent archaic varieties. A pertinent question is whether the conclusions made for the available text types will hold if we use data representing other registers.

Challenge 3. *Low representativity: Translationese and educationalese*. The language of corpora can differ from naturally produced language, due to the influence of the source language on translations in parallel corpora, or inclusion of non-naturalistic data, such as textbook examples in some Universal Dependencies corpora. This can create undesirable biases. For example, Östling (2015) found that Romance languages in his corpus (Spanish, Catalan, Portuguese, French and Italian) have predominantly adjective–noun word order. The reason is that the corpus contains translations of the New Testament, in which this order is preferred. Although this does not necessarily mean that the data are unusable, one should be aware of the risks involved in using them for a particular research question.

Challenge 4. *Low efficiency*. Corpus mining is extremely time-consuming, especially in cases where there is no annotation and alignment. Corpora rarely have glosses or translations into some major language, which often makes it difficult to spot problematic issues and interpret the results. On a positive note, computational linguists are developing automatic solutions and tools in order to make corpus analysis more practically feasible (e.g. Östling 2015; Straka and Straková 2017).

Challenge 5. *Noise and loss of information*. Corpus data are usually noisy. In particular, automatic annotation tools produce a lot of errors, from tokenization to syntactic parsing. Also, text sources can contain spelling and punctuation mistakes, especially web-based corpora. Human annotators can make mistakes, as well. In some cases, annotation decisions are extremely difficult to make, for example, identification of clause boundaries in spoken corpora or coding of covert arguments. Linguistic categories are by nature gradient, so any annotation choice leads to a loss of information. This is a general problem of language description. A solution would be to reflect that genuine uncertainty in coding and allow the researcher to make his or her own decisions.

Challenge 6. *Low comparability*. Different corpora can have different annotations procedures, lists of POS tags, morphological features or syntactic dependencies (e.g. Osborne and Gerdes 2019). Even in the Universal Dependencies project, different corpora can have different solutions for functionally similar words. For example, the possessive pronouns in the English corpus EWT have the tag PRON (pronouns), whereas in the Czech corpus PDT and the Russian corpus SynTagRus they are annotated as DET (determiners). All this slows down the workflow and increases the risk of obtaining misleading results.

Challenge 7. *Word as a unit of analysis*. One of the most important questions is what to count as a word. As argued by Haspelmath (2011), there are no necessary and sufficient criteria for defining the word as a cross-linguistic comparative concept. Therefore, “linguists should be very careful with general claims that make crucial reference to a cross-linguistic ‘word’ notion” (Haspelmath 2011: 31). The orthographic approach, where a word is just a character string between two spaces or punctuation marks, is often taken for granted in corpus-based studies. Corpus linguists sometimes compare languages using metrics based on orthographic words, e.g. dependency distances (Liu 2010). These results can be difficult to interpret. Yet, to be fair, it is still unclear what a valid alternative would be, also in qualitative typological studies. Moreover, some corpora (e.g. Universal Dependencies) allow us to choose between different approaches with the help of special coding information about multiword units and contractions, and to compare the results based on different methods, as will be shown in the next section.

## 11 A case study of analyticity indices

This section provides a small case study which illustrates how corpora can be used for language comparison. It also shows the relevance of register variation and the problems with defining the word as the unit of analysis, which were mentioned in the previous section. We will focus on analyticity index (i.e. the proportion of function words in the total number of words), which was discussed in Section 5.1. Will the index be stable if we take different text types? This problem was already clear to Greenberg (1960), who computed the index of syntheticity for English and German written texts of varying style in order to make sure that the style does not introduce biases. The indices were remarkably similar, however. In contrast, Szmrecsanyi’s (2009) work on different stylistic, as well as geographic and diachronic, varieties of English demonstrates clearly that English is not homogeneous with regard to analyticity and syntheticity.

The other problem is what to count as a word. This issue is crucial for computing analyticity, syntheticity and similar typological indices because they contain the number of words in their denominator. It is interesting that Greenberg was quite relaxed about it:

In certain cases there seems to be no good reason for the choice of one alternative over the other from this point of view, and a purely arbitrary choice was made since some basis of decision had to be reached. It may be of some comfort to note that the theoretically wide range of choice of definitions for certain units only bore on decisions for a relatively small proportion of difficult instances. (Greenberg 1960: 188)

This statement, however, has not been checked systematically on corpus data.

In our case study, we compare Arabic, English, Finnish, French, German, Indonesian, Russian and Turkish, using different text types and different approaches to defining the word. The corpora represent three text types: online news, Wikipedia and film subtitles, which serve as a proxy for informal conversations (Levshina 2017b). The news and Wikipedia corpora, each consisting of 30,000 sentences, are taken from the Leipzig Corpora Collection (Goldhahn et al. 2012). The film subtitles represent five films from ParTy, a corpus of online film subtitles (Levshina 2015). The texts are annotated with UDPipe, a toolkit for Universal Dependencies annotation software (Straka and Straková 2017), implemented in the R package *udpipe* (Wijffels et al. 2018), which provides tokenization, lemmatization, part-of-speech annotation and syntactic parsing.

As far as the word definition is concerned, there are several factors to take into account. First, one can ask whether diverse complex expressions like compound nouns (e.g. *art history*), phrasal verbs (e.g. *sign up*) and proper names (e.g. *New York*) should be counted as one or two words. Another case is complex prepositions and conjunctions, e.g. *in spite of* and *as well as.* Why should that matter? If, for example, the expression *art history* is counted as two words in English, but its equivalent in German, *Kunstgeschichte*, is counted as one word, this will create a bias. Moreover, there are spelling variants even within one language, e.g. *school teacher* and *schoolteacher*. Another question is how to analyse diverse phonologically unified combinations of two words that involve function words, such as auxiliary and negation contractions (e.g. *I’ll, can’t*), case particles (e.g. *-no* and *-wa* in Japanese) or pronominal clitics (e.g. *l’ai* or *m’appelle* in French).

To take these issues into account, we compare three approaches to defining the word:

1. The orthographic approach. Words are strings separated by white spaces or punctuation marks (except for apostrophe or hyphen). For example, contractions like English *I’ll* and French *l’ai* are treated as one word, while *as soon as* contains three words.
2. The “lumping” approach. Multiword units are treated as one word. In the UD annotation, these are the combination of strings connected by the following dependencies: “compound”, which describes compounds like *noun phrase;* “fixed”, which combines expressions like *in spite of*; and “flat”, which is used to analyse names like *Angela Merkel.*17See more at https://universaldependencies.org/u/overview/specific-syntax.html#multiword-expressions. If a word is not in a multiword unit, the orthographic approach is followed.
3. The “splitting” approach. In addition to orthographic tokens, some UD corpora provide information about their morphemic components. An example is German *zum*, which is a merge of the preposition *zu* and the definite article in the dative case *dem.* Similarly, Arabic conjunctions (e.g. *wa* “and”), are written as a prefix, but are analysed as separate words. The Turkish suffix *-ki*, which forms absolute possessive (e.g. *babamınki* “my dad’s”), is treated as a separate unit, as well. In these examples, both the orthographic and morphemic representations are available. Some corpora only provide the components, even if they are written as one orthographic word. For example, English contractions like *I’ll* and *don’t* are split into two tokens. In the splitting approach, we use this information, counting every component wherever possible. If a word cannot be split into smaller units, the orthographic approach is followed.

From every corpus, we took 100 random samples of 1,000 words (tokens) defined as above. Punctuation marks, numbers, symbols and unknown words were excluded. For each sample of 1,000 words, the analyticity index was computed. We relied on the Universal Parts of Speech (UPOS) in order to distinguish function words from content words.18See more information at https://universaldependencies.org/u/pos/index.html. If a word had one of the UPOS tags below, it was counted as a function word, and 1 was added to the sum score:

– determiners (DET),
– pronouns (PRON),
– auxiliaries and copulas (AUX),
– adpositions, including case particles (CASE),
– particles (PART),
– subordinate (SCONJ) and coordinate (CCONJ) conjunctions.

For all other UPOS tags, 0 was added. There are many cases in orthographic and lumping approaches when a word is counted as one unit, but consists of two, one of which has a function word tag, and the other has a content word tag. For example, *s*’*appeler* has two tags, PRON and VERB. In that case, 0.5 was added. Finally, the sum score was divided by the sample size (1,000). The data are available in Supplementary Materials.

**Figure 3:**
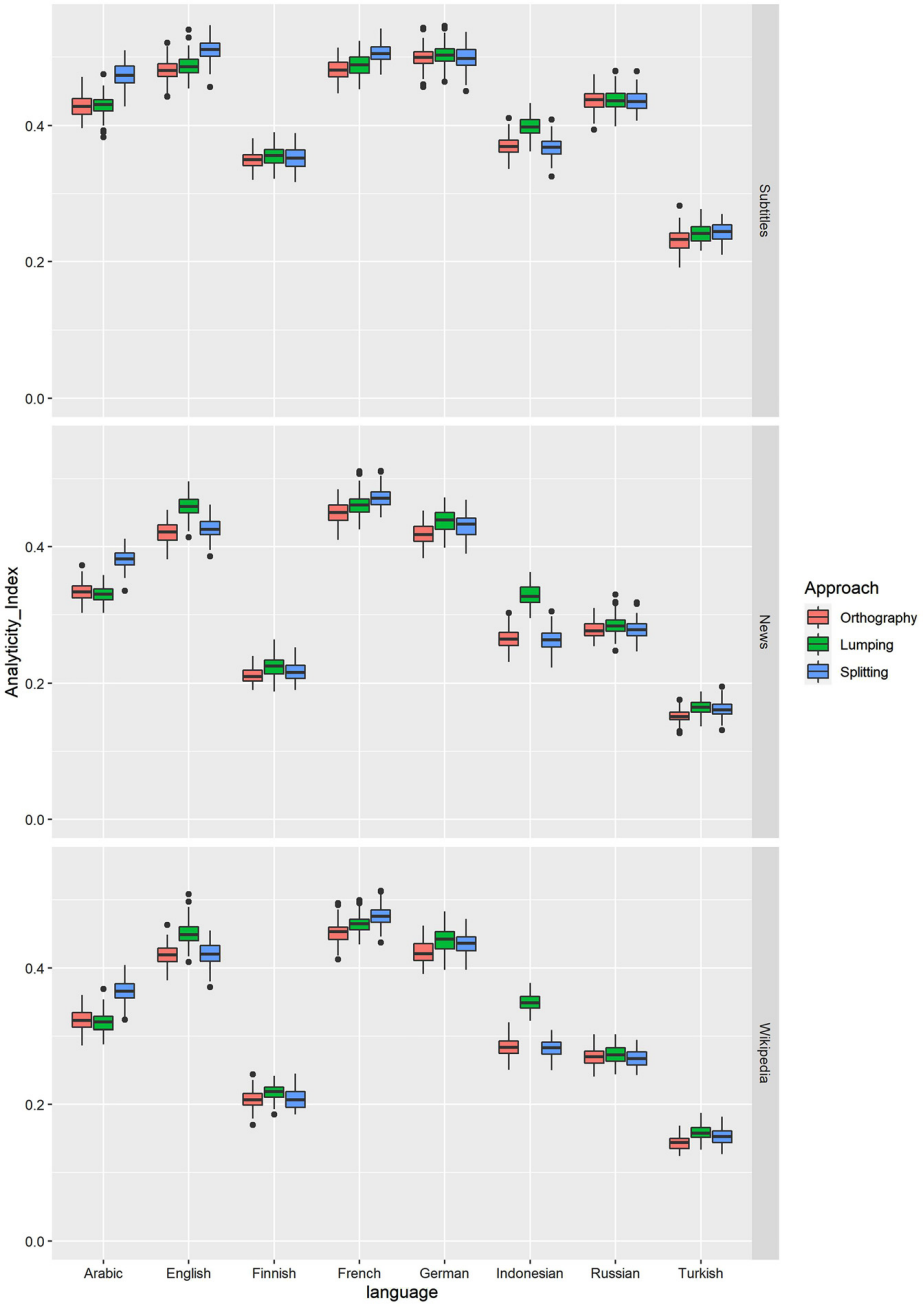
Analyticity indices in samples of 1,000 tokens based on Universal Dependencies POS-tags.

**Figure 4:**
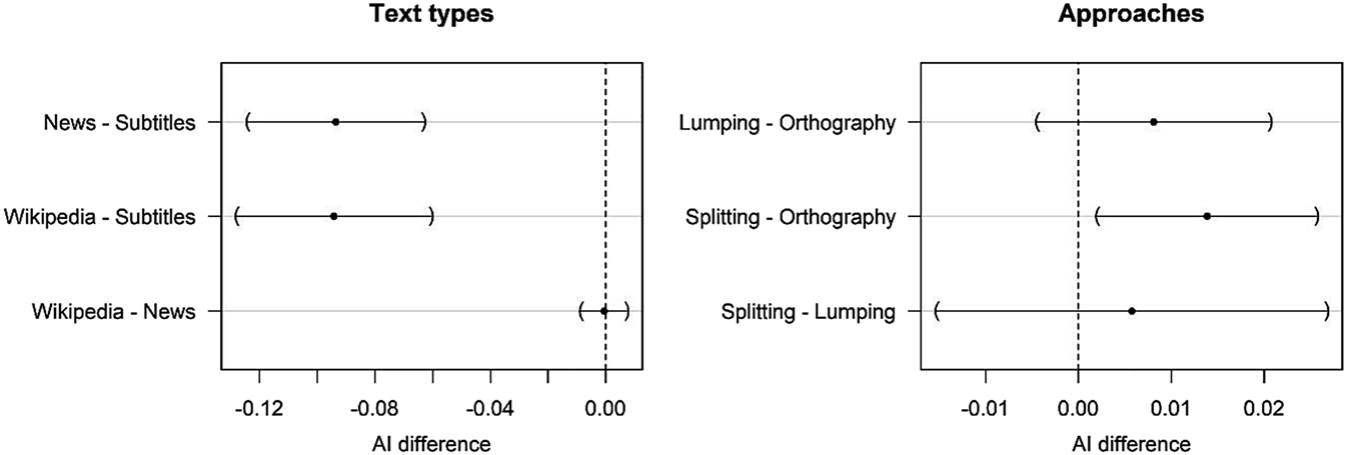
Differences between pairs of text types and approaches.

The results, which are displayed in Figure 3, demonstrate that the indices vary a lot, depending on the text type. The film subtitles, which represent informal language, have higher analyticity indices than the written registers. A mixed-effect linear regression analysis reveals significant differences between the subtitles and the other text types.19The model structure was Analyticity_Index ∼ (1 + text_type + Approach|language) + text_type + Approach + text_type:Approach. The maximum likelihood criterion was used for fitting. Tukey’s correction was used for multiple comparisons. The differences between all pairs of registers with 95% confidence intervals are displayed in Figure 4 (left). The difference between the news and Wikipedia articles is very close to zero. They are not significantly different. Note that Greenberg (1960) compared only written texts, when he made his conclusion about the similarity of different texts in English and German. However, the ranking of the languages is roughly the same for the written texts and the subtitles. An exception is the position of Russian and Indonesian: Russian subtitles are more analytic than Indonesian ones, but the other texts show the opposite.

Let us now turn to the second question related to how we define the word. In English and Indonesian, the lumping strategy leads to higher analyticity in the Wikipedia and news texts, where Noun + Noun compounds abound. In Indonesian, this difference is also observed in the subtitles. In Arabic and less strongly in French, the splitting strategy increases the index. This is due to the fact that many function words in both languages are merged orthographically, which increases analyticity. How we define the word is less important in Finnish, German, Russian and Turkish, where the differences are small. A test of multiple comparisons based on the mixed-effects model shows that the differences between the approaches are small (see Figure 4, right). At the same time, the difference between the splitting and orthographic approaches is statistically significant. To what extent these differences are significant practically and theoretically is a question open for discussion.

Thus, we find a lot of variation depending on text types. This means that we need to be careful when comparing languages represented by different genres. As for the definition of the word, the differences are less striking, but statistically significant. On a more optimistic note, we can conclude that the rankings do not change very dramatically either with the approach, or with the text type, provided that we stick to one text type and to one approach. There are highly analytic languages (English, German and French), followed closely by Arabic, Indonesian and Russian, and then by weakly analytic Finnish and finally Turkish.

To conclude, there are many restrictions and challenges involved in using corpus data for typological purposes. It is always advisable to compare different text types and different approaches. Yet, when used with caution, corpus data can help us get new insights into linguistic diversity and universals.

## Supplementary Material

Supplementary Material Details

## Appendix

**Table 1:** Greenberg’s corpus-based indices for morphological typology.

Index	Meaning	Illustrations
index of synthesis	the ratio of the number of morphemes to the number of words	*sing-s* has 2 morphemes
index of agglutination	the ratio of agglutinative constructions to the number of morpheme junctures	*kind-ness* is an agglutinative juncture; *dep-th* is a non-agglutinative juncture
compounding index	the ratio of the number of root morphemes to the number of words	*black-bird* has two root morphemes
derivational index	the ratio of derivational morphemes to words	*lion-ess* has one derivational morpheme (*-ess*)
gross inflectional index	the ratio of inflectional morphemes to words	*sing-s* has one inflectional morpheme (*-s*)
prefixial index	the ratio of prefixes to the number of words	*re-make* has one prefix
suffixial index	the ratio of suffixes to the number of words	*sing-s* has one suffix
isolational index	the ratio of grammatical dependencies without inflectional morphemes to the number of grammatical dependencies	*nice person* has no features expressed by inflectional morphemes
pure inflectional index	the ratio of grammatical relationships (features) expressed by “pure” inflections (i.e. not concord) to the number of grammatical dependencies	*he walk-ed* has one feature (past tense) formally expressed by a morpheme
concordial index	the ratio of formally expressed concord features to the number of grammatical dependencies	*he sing-s* has two concord features (3^rd^ person, singular) formally expressed by a morpheme
